# Barriers to investigate and to report nosocomial outbreaks to health authorities in São Paulo, Brazil: a mixed method approach

**DOI:** 10.1186/2047-2994-4-S1-P234

**Published:** 2015-06-16

**Authors:** ALP Maciel, BA Carvalho, MC Padoveze

**Affiliations:** 1 School of Nursing, Universidade de São Paulo, Sao Paulo, Brazil

## Introduction

Nosocomial outbreak investigations (NOI) may provide relevant insights into the field of healthcare-associated infection. Nevertheless, in the last ten years only a quarter of all published NOI were reported to the health authorities (HA) in São Paulo State, Brazil (SPS).

## Objectives

We aimed at to identify barriers to investigate and to report NOI to the HA in São Paulo.

## Methods

A mixed methods approach was performed in a convergent parallel design. The quantitative branch of the study was a statewide survey by means of an electronic questionnaire. The qualitative branch was carried out by means of focus groups (FG). Infection control practitioners (ICP) working in SPS were recruited. Data were processed individually in a descriptive analysis (electronic survey) and a content analysis (FG).

## Results

ICP enrolled were 87 and 22 respectively in the electronic survey and FG. A similar proportion of nurses (60%) and physicians (40%) were included in both branches of the study. Data from the survey and FG were convergent regarding to: i) although most ICP believe themselves with enough knowledge on NOI, they find difficult translate this knowledge into practice; ii) ICP perception is that sufficient human and material resources are present in hospitals, but overall there is weak planning in infection control activities; iii) ICP do not feel supported by hospital managers; iv) ICP know the channels to report outbreaks to HA (84%), but they perceive it as meaningless; vi) ICP don’t report to HA because they get concerned about potential punishment (64%) or institutional image damage (52%). There were two divergent results regarding to: i) laboratory support: In the survey ICP informed to have good laboratory support (59%), however, in FG participants complained about that; ii) interaction with HA: in the electronic survey participants referred good interaction (50%) and no punishment (84%) related to HA, but in FG they declared a poor interaction.

## Conclusion

Our results showed that barriers to NOI and reporting to HA are knowledge, skills and hospital manager support. HA should overcome these barriers by rebuilding its strategies to approach health care services as well as delivering translational educational programs to support improvement NOI skills.

## Disclosure of interest

None declared.

